# “Universal” Antimicrobial Combination of Bacitracin and His_6_-OPH with Lactonase Activity, Acting against Various Bacterial and Yeast Cells

**DOI:** 10.3390/ijms23169400

**Published:** 2022-08-20

**Authors:** Aysel Aslanli, Maksim Domnin, Nikolay Stepanov, Elena Efremenko

**Affiliations:** Chemical Faculty, Lomonosov Moscow State University, Lenin Hills 1/3, 119991 Moscow, Russia

**Keywords:** bacitracin, hexahistidine-containing organophosphorus hydrolase, combination, molecular docking, quorum quenching, bacteria, yeast

## Abstract

The effect of Bacitracin as an antibiotic acting against Gram-positive bacterial cells was evaluated in combination with hexahistidine-containing organophosphate hydrolase (His_6_-OPH), possessing lactonase activity against various *N*-acylhomoserine lactones produced by most Gram-negative bacteria as quorum-sensing molecules. The molecular docking technique was used to obtain in silico confirmation of possible interactions between molecules of His_6_-OPH and Bacitracin as well as the absence of a significant influence of such interactions on the enzymatic catalysis. The in vitro experiments showed a sufficient catalytic efficiency of action of the His_6_-OPH/Bacitracin combination as compared to the native enzyme. The notable improvement (up to 3.3 times) of antibacterial efficiency of Bacitracin was revealed in relation to Gram-negative bacteria when it was used in combination with His_6_-OPH. For the first time, the action of the Bacitracin with and without His_6_-OPH was shown to be effective against various yeast strains, and the presence of the enzyme increased the antibiotic effect up to 8.5 times. To estimate the role of the enzyme in the success of His_6_-OPH/Bacitracin with yeast, in silico experiments (molecular docking) with various fungous lactone-containing molecules were undertaken, and the opportunity of their enzymatic hydrolysis by His_6_-OPH was revealed in the presence and absence of Bacitracin.

## 1. Introduction

Today, the search for an effective solution to the problem of resistance in microorganisms and the prevention of the formation of multicomponent microbial highly concentrated populations of cells resistant to various influences and conditions of existence, that are resistant to various influences and conditions of existence in a quorum state, is still relevant (www.who.int/news-room/fact-sheets/detail/antimicrobial-resistance, accessed on 18 August 2022). Quorum Sensing (QS) is one of the obvious reasons for the formation of resistant microbial biosystems [1]. The use of Quorum Quenching enzymes (QQE), hydrolyzing the so-called QS molecules already synthesized by microorganisms, which trigger cell restructuring processes leading to changes in their metabolism and biochemical composition, is of increasing scientific and practical interest, as it is very effective [2,3]. Especially in the case of combining such enzymes with widely used antibiotics, to which resistance is formed [3,4,5], as well as with antimicrobial polypeptides (AMPs) of natural origin, which can form an alternative to traditional antimicrobial blockbusters [6]. Such combinations of QQ and antimicrobials can either reduce the effective dose of the latter or increase the effect of a typically-used dose.

**Figure 1:**
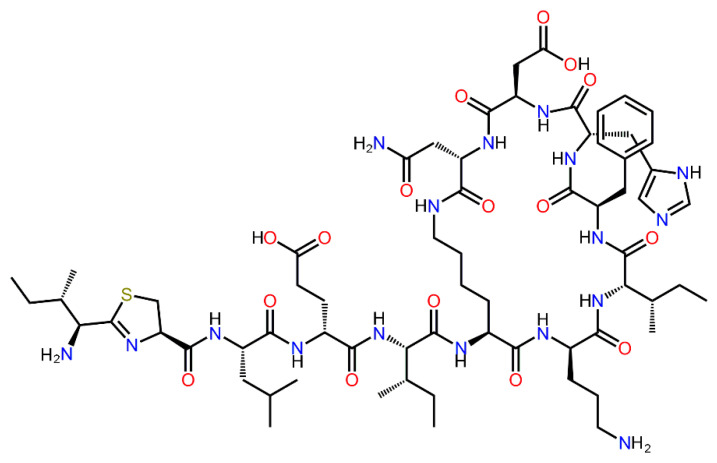
Structure of Bacitracin A.

Today, it can be noted that there is maximum interest in AMPs with a wide spectrum of action in relation to Gram-negative (G(−)) and Gram-positive (G(+)) bacterial cells [5,6,7]. For this purpose, an environmental search and organic synthesis of new AMPs with an extended spectrum of action is being conducted. Moreover, modification of already-known AMPs with well-studied characteristics is being carried out in order to also expand the list of objects of influence for them [6,8]. Such natural AMPs include Bacitracin synthesized by bacterial cells *Bacillus licheniformis* and *Bacillus subtilis*, which has pronounced antimicrobial activity against G(+) bacterial cells and acts by inhibiting the synthesis of their cell walls, in particular, peptidoglycan [7,9,10,11,12]. In fact, Bacitracin is a mixture of related cyclopeptides, and Bacitracin A (Figure 1) is the most active antimicrobial form with molecular weight 1422.7 Da, which consists of 12 amino acid residues, four of which are D-derivatives [7]. As a result, Bacitracin has nephrotoxicity [10], and therefore is not used by injection. However, since the D-amino acid residues in Bacitracin provide it with “protection” from degradation by proteases capable of cleaving peptide bonds between L-amino acid residues [10], interest in this AMP and its use remains, and today its low concentrations are widely used even as an effective feed additive to maintain healthy growth of agricultural animals. After oral application of Bacitracin to various animals it is hardly absorbed from the gastro-intestinal tract, and its distribution to organs and tissues is negligible since approximately 98% of Bacitracin is usually excreted [11]. Since then, Bacitracin has appeared to be more attractive for the application as means for the antimicrobial treatments of various surfaces, including skin, of waste water and manure, etc.

It should be noted that Bacitracin has an original mechanism of action: it modifies the substrate of the pyrophosphatase, lipid C55-isoprenyl pyrophosphate (C-PP), in the presence of Zn^2+^ ions or other divalent cations [13]. As a result, C_55_-isoprenyl phosphate (C_55_-P) is not formed from C_55_-PP, which, in turn, is a membrane carrier of *N*-acetylglucosamine (NAG) involved in the construction of the bacterial cell wall, specifically peptidoglycan. Hence, the high efficiency of Bacitracin’s action is precisely in relation to G(+) bacterial cells.

Interestingly, despite the differences in the structure of the cell wall of G(+) and G(−) bacterial cells, as well as in pro- and eukaryotic cells of microorganisms (for example, bacteria and yeast), all of them are characterized by the structural presence of NAG: chitin is the main component of the cell wall of fungi, and, in fact, peptidoglycan is the most important part of the bacterial cell wall, as well as pseudopeptidoglycan (also known as pseudomurein) which presents in archaea cells [14]. Consequently, it can be expected that a violation of the NA molecule transfer process to the assembly site of the corresponding polysaccharides may lead to a slowdown, or even complete inhibition, of the synthesis of the cell wall of these microorganisms. If such a study with Bacitracin was carried out with respect to archaea [15], then we could not find such information with respect to yeast cells.

Taking into account the above, in this work we investigated the effect of Bacitracin as an antimicrobial substance on different yeast cells. Moreover, in this study we decided to compare the effectiveness of Bacitracin in combination with such QQE as hexahistidine-containing organophosphate hydrolase (His_6_-OPH), which exhibits lactonase activity against various *N*-acyl homoserine lactones, which are QS inducer molecules of most G(−) bacteria [3].

We decided to test this combination of His_6_-OPH/Bacitracin in parallel both on yeast and against G(−) bacterial cells, since we noted the interest in the possibility of expanding the spectrum of action of Bacitracin above. The same combination was studied in relation to some G(+) bacterial cells, to compare the Bacitracin action effectiveness alone and in combination with His_6_-OPH.

The effectiveness of using computer modeling methods in studying interactions between His_6_-OPH and various antimicrobial agents to obtain reliable and reproducible results at the first stage of the study was demonstrated earlier in the development of active antibacterial drugs [3,4].

In this work, the molecular docking method was also applied, which initially allows choosing the most suitable models of molecular interactions and taking into account the presence of a solvent in the reaction medium at different pH values. Obtaining models confirming possible interactions between His_6_-OPH and bacitracin molecules, and the absence of the likelihood of a significant effect of such interactions on enzyme activity, was necessary as a first step in studies of the catalytic and antimicrobial efficacy of the His_6_-OPH/Bacitracin combination.

The effectiveness of using computer modeling methods in studying interactions between His_6_-OPH and various antimicrobial agents to obtain reliable and reproducible results at the first stage of the study was demonstrated earlier in the development of active antibacterial drugs [3,4]. In this work, the molecular docking method was also applied, which initially allows choosing the most suitable models of molecular interactions and taking into account the presence of a solvent in the reaction medium at different pH values. Obtaining models confirming possible interactions between His_6_-OPH and Bacitracin molecules, and the absence of the likelihood of a significant effect of such interactions on enzyme activity, was necessary as a first step in studies of the catalytic and antimicrobial action efficacy of the His_6_-OPH/Bacitracin combination.

## 2. Results

### 2.1. Computational Modeling of His_6_-OPH/Bacitracin Interactions

To obtain a model of the interaction of a Bacitracin molecule with a dimeric enzyme molecule and to study the characteristics of this interaction, the molecular docking method was used.

**Figure 2:**
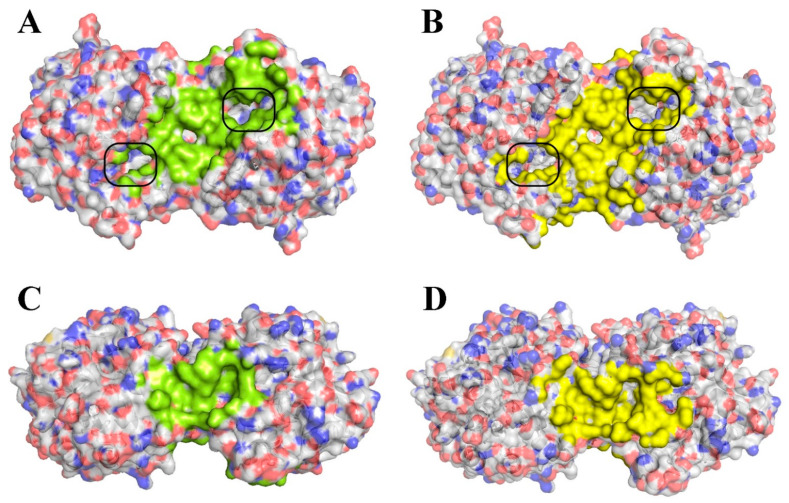
Front view (**A**,**B**) and top view (**C**,**D**) of supposed localization of Bacitracin molecules on the surface of His_6_-OPH dimer at pH 7.5 (**A**,**C**) and 10.5 (**B**,**D**). The atoms located within 4Å of any Bacitracin atom and the corresponding molecular surface, are colored green and brown at pH 7.5 and 10.5, respectively. The active sites of the enzyme dimer are marked by black boxes.

Molecular modeling was carried out at two pH values of the medium: 7.5 (as the closest to physiological conditions) and 10.5 (corresponding to the maximum catalytic activity of the enzyme [16]). Further, the characteristics of the interactions of the enzyme and the antibiotic in the combination His_6_-OPH/Bacitracin were analyzed. In particular, the localization of Bacitracin molecules on the surface of the enzyme dimer, the size of the areas occupied by the molecules of this AMP on the entire surface of the enzyme and near the active sites of the His_6_-OPH dimer, and the values of the interaction energy (affinity–i.e., an estimate of the binding energy within electrostatic and hydrophobic interactions, and the hydrogen bonding between ligand and receptor) between the molecules in question (Table 1, Figure 2) were controlled.

It was found that Bacitracin has minimal localization near the active sites of the enzyme dimer, while the availability of the enzyme’s catalytic sites for its typical substrates is poorly screened when using the resulting His_6_-OPH/Bacitracin combination in possible catalytic reactions. In this regard, it was concluded that a small negative effect of Bacitracin in the reaction medium on the manifestation of the catalytic activity of His_6_-OPH should be expected.

It should be noted that rather high total calculated values of affinity (interaction energy) were obtained between functional groups located on the surface of the enzyme molecule and Bacitracin at both studied pH values (Table 1), which indicated in favor of the fact that such interactions should lead to the formation of non-covalent complexes, which are characteristic of His_6_-OPH at interaction with other AMPs [4,5].

### 2.2. Catalytic and Physical-Chemical Characteristics of His_6_-OPH in the Absence and Presence of Bacitracin

**Figure 3:**
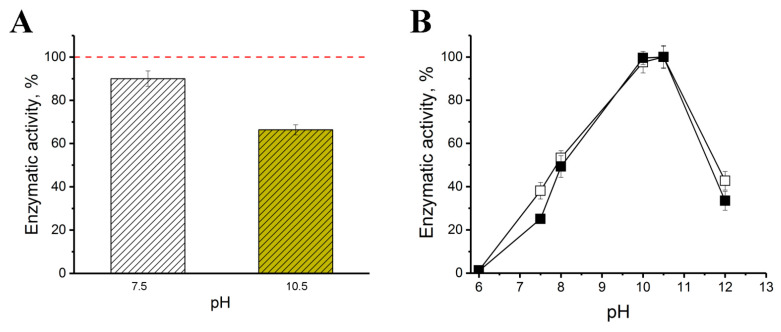
(**A**) Enzymatic activity of His_6_-OPH/Bacitracin combinations, formed at pH 7.5 and 10.5 after their exposition for2 h at 8 °C in water solution, when the initial protein concentration used for the combination obtaining was 1 mg/L. The red dotted line corresponds to the initial level of enzyme activity assessed as 100%. (**B**) pH-dependence of His_6_-OPH activity estimated in the presence (■) and absence (**☐**) of Bacitracin, when the His_6_-OPH/Bacitracin combination was obtained at pH 7.5.

In order to determine at which of the two pH values (7.5 or 10.5) the combination of His_6_-OPH/Bacitracin is the most appropriate for use in practice, from the point of view of stabilizing the catalytic activity of the enzyme, appropriate samples of the combination under study were obtained. Further, these samples of His_6_-OPH combinations with Bacitracin, formed at pH 7.5 and 10.5, were exposed at 8 °C for 2 h, then used for hydrolysis of a typical substrate of this enzyme and the residual activity was determined (Figure 3).

It was found that the residual activity of the studied combination formed at pH 7.5 was 1.4 times higher than that obtained at pH 10.5 (Figure 3A). Based on this, in further studies, it was decided to use the combination of His_6_-OPH/Bacitracin, formed precisely at pH 7.5. The study of the pH of the optimum action of the enzyme in the presence of Bacitracin (the combination was obtained at pH 7.5) and in the absence of AMP (Figure 3B) confirmed the fact that cyclopeptide actually minimally affects the catalytic properties of the enzyme. The optimum action of the combination of the resulting His_6_-OPH/Bacitracin remains the same as that of the native enzyme.

Further, the main catalytic characteristics of the His_6_-OPH/Bacitracin combination were determined (Table 2), when the combination was obtained at pH 7.5 and the investigation was conducted at optimal pH value for enzyme action. For comparison, similar studies were conducted in parallel with the native enzyme.

The K_m_ value, which was determined for the combination of His_6_-OPH/Bacitracin, was 1.5 times higher than for the native enzyme (Table 2). This fact testified in favor of the fact that, probably, the presence of Bacitracin still partially shielded the availability of the active site of the enzyme for the substrate.

However, the catalytic constant (V_max_/E_0_) turned out to be better than that of the native enzyme, providing a conformation with a higher rate of hydrolytic reaction with the substrate that has already entered the active site of His_6_-OPH and formed an enzyme-substrate complex. Interestingly, we have already observed a similar effect of some enzyme partners such as Temporin A and Indolicidin, which are also small AMP molecules, on the catalytic characteristics of His_6_-OPH [4], and the results obtained in this study were no longer a surprise for us.

The value of the efficiency constant of the enzyme in the combination of His_6_-OPH/Bacitracin (V_max_/(E_0_ × K_m_) turned out to be comparable with the same parameter established for the native enzyme. The data are generally consistent with the results of molecular docking: the catalytic activity of the enzyme in the presence of Bacitracin relative to the native enzyme did not change notably.

**Figure 4:**
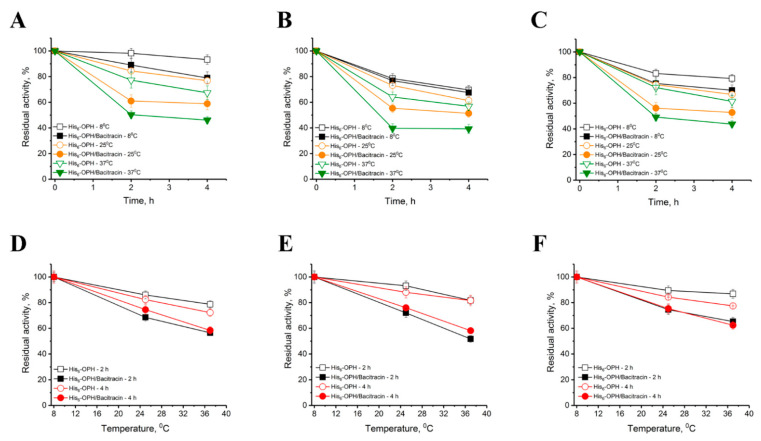
Kinetics of thermoinactivation of His_6_-OPH exposed in various concentrations (1 mg/L (**A**), 10 mg/L (**B**) and 20 mg/L (**C**) at 8, 25 and 37 °C at pH 7.5 in the absence and presence of Bacitracin. The activity of the native enzyme under the same conditions was assumed to be equal to 100%. Trends of thermoinactivation of His_6_-OPH activity in the absence and presence of Bacitracin at concentration 1 mg/L (**D**), 10 mg/L (**E**) and 20 mg/L (**F**) within 2 and 4 h are presented as a function of temperature. The activity at 8 °C was taken as 100%.

Further, the influence of the temperature and concentration of the His_6_-OPH/Bacitracin combination, varying by protein in an aqueous medium, but maintaining a constant ratio between the enzyme and the antibiotic, on the enzymatic activity of the combination itself and the stability of its catalytic properties was studied (Figure 4).

The results obtained showed that the residual activity of the enzyme combination with AMP at the initial protein concentration of 10 mg/L after 4 h of exposure at a temperature of 8 °C was ~ 70% of the initial level, and it was about ~ 60% at 25 °C and 40% at 37 °C. Obviously, with an increase in the concentration of the combination in solution by the protein, its thermal stability slightly improved. It was found that the presence of Bacitracin leads to a more noticeable kinetics of loss of enzyme activity in the range of concentrations studied, however, the final results of activity decrease were similar to each other after 4 h. The maximum level of residual enzyme activity was observed at 8 °C. These data are certainly important to take into account when the possible practical application of combinations of the enzyme and Bacitracin is expected.

### 2.3. Antimicrobial Activity of Bacitracin alone or in Combination with His_6_-OPH

When studying the effect of the presence of His_6_-OPH on the effectiveness of Bacitracin action, the antimicrobial activity was determined in samples with various microorganisms, among which there were both G(−) bacterial cells (*Escherichia coli* DH5a, *Agrobacterium tumefaciens* B-8833) and G(+) bacterial cells (*Rhodococcus ruber* AC-1513D, *Bacillus subtilis* B-522), as well as cells of various yeasts (*Candida* sp.Y94, *Saccharomyces cerevisiae* (Vintage white, EnartisFerm, San Marino, Italy), *Cryptococcus albidus* Y-16, *Pachysolen tannophilus* Y-475, *Kluyveromyces marxianus* Y-18, *Torulopsis* spp. Y-276 and *Trichosporon beigelii* Y-253) in the absence and presence of His_6_-OPH (Table 3). The antimicrobial effect of the AMP and its combination with the QQE was evaluated by reducing the level of intracellular ATP concentration in the studied samples of cell suspensions (Table 3).

G(+) bacterial cells and yeast *Cryptococcus albidus* were the only exceptions in these studies, for which the use of His_6_-OPH/Bacitracin combination did not lead to a decrease in IC_50_. In all other cases, it was found that in the presence of the enzyme, a decrease in the value of IC_50_ values was observed both in relation to bacterial cells and yeast cells. For example, the most significant decrease in inhibitory concentration was observed for bacteria *Pseudomonas* sp. and *Agrobacterium* sp. (~3.2–3.3 times) and yeast *Pachysolen tannophilus* (~8.5 times).

For many of the studied cultures of microorganisms, there was also a significant decrease in the values of inhibitory concentrations (3.2–3.9 times) necessary for the manifestation of effective antimicrobial activity for the combination of the enzyme with AMP in comparison with a single Bacitracin.

### 2.4. In Silico Estimation of Possible Hydrolysis Catalyzed by His_6_-OPH of Lactone-Containing Molecules Synthesyzed by Fungous (Including Yeast) Cells

The results obtained with yeast cells in the study of the antimicrobial activity of Bacitracin in the combination with His_6_-OPH allowed to assume that the success of the function of the composition indicated that His_6_-OPH probably exhibits lactonase activity not only against lactone-containing molecules of the quorum of G(−) bacterial cells, but also and in relation to various lactones synthesized by different fungi, including yeast. Among such lactone-containing molecules, secondary metabolites are known, which are used by fungi as metabolism regulating and fighting agents (mycotoxins) and QS signaling molecules [17,18,19,20].

The assumption about the possible hydrolytic effect of His_6_-OPH on yeast lactones was made based on the known properties of this enzyme, for which we had previously established the ability to effectively hydrolyze various lactone-containing mycotoxins (zearalenone and patulin) synthesized by fungi as their own means of protection against competitors using the same substrates [21].

To confirm the validity of our assumption about the possible hydrolysis of lactone-containing fungal/yeast molecules under the action of His_6_-OPH, an in silico study was conducted in which molecular docking of various lactone-containing fungal molecules to the surface of the His_6_-OPH dimer was performed (Table 4 and Table S1, Figures S1 and S2).

**Figure 5:**
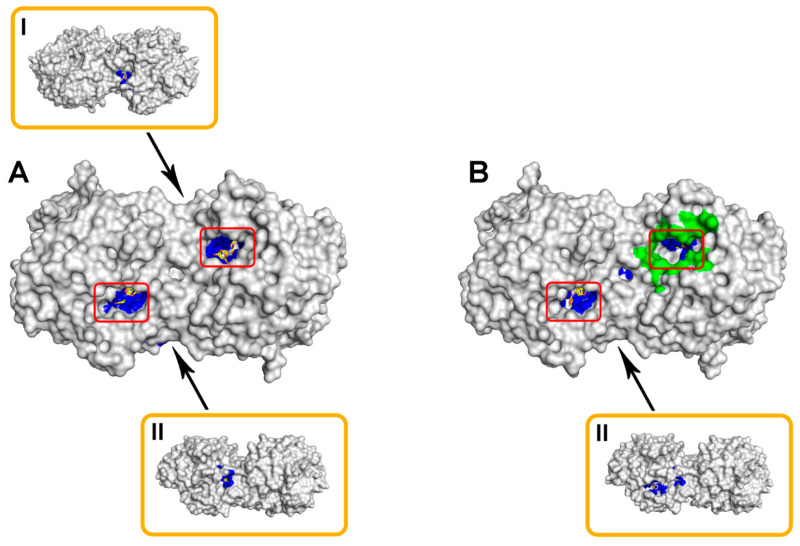
Computationally-predicted localization of lactone molecules (colored yellow) on the surface of dimeric His_6_-OPH molecule (colored grey) in the absence (**A**) and in the presence (**B**) of Bacitracin molecule (colored green). The molecular surface corresponding to atoms located within 4 Å of any lactone atom is colored blue. The entrances to the active sites of His_6_-OPH dimer are highlighted with red boxes. Orange boxes show a His_6_-OPH dimeric molecule, when viewed above (**I**) and below (**II**) (relative to the active site localization).

At the same time, such computer modeling was performed in the presence and absence of Bacitracin on the surface of the enzyme (Table 4, Figure 5). Particular attention was paid to the localization of fungal lactone-containing molecules in the active site of the enzyme, as a necessary condition for the course of the hydrolytic reaction we propose.

The results of molecular docking showed that, regardless of the presence or absence of Bacitracin in the region of the active sites of the His_6_-OPH dimer, for three compounds from among the studied, which are most often found in the yeast medium (γ-butyrolactone, γ-heptalactone, and γ-decalactone), their localization in the catalytic sites of the enzyme was visible (Table 4). Such data suggest that these molecules have the maximum predisposition to enzymatic hydrolysis, which indirectly confirms our initial assumptions.

## 3. Discussion

The use of the molecular docking method allowed, in silico, to simulate the interaction of His_6_-OPH molecule with Bacitracin, and it was established that this AMP can be localized both near the active sites of the dimer of this enzyme and interact with other parts of the His_6_-OPH surface (Figure 2). At the same time, the area that Bacitracin can occupy near the active site has an insignificant value (Table 1), whereas in general, Bacitracin molecules on the surface of the enzyme can occupy 10–14%, depending on the pH of the medium. It should be noted that an insignificant number of AMP molecules in the region of the active site of the enzyme was noted because the region of the active site of His_6_-OPH itself, as it was established earlier, has a negative charge [12], and the molecules of Bacitracin itself have the same negative charge. Comparison of the influence of the location of various peptide molecules on the surface of other enzymes on the catalytic activity of the latter (Table 5) showed that binding directly to the active site residues of the enzyme leads to inhibition of its catalytic activity. Moreover, the higher the degree of blocking of the entrance to the active site of the enzyme by peptide docked, the more significant inhibition is observed further in wet experiments.

Nevertheless, the small number of Bacitracin molecules that appear near the active sites of His_6_-OPH obviously affect the binding of the enzyme to its typical substrate, which leads to an increase in the Michaelis constant (Table 2). The configuration of the enzyme molecule formed and stabilized (Figure 3) under the influence of interactions with Bacitracin molecules. That leads to increase in the catalytic constant value of the action of the His_6_-OPH/Bacitracin combination, which in total leads to insignificant changes in the catalytic efficiency of His_6_-OPH in combination with AMP compared with the same parameter characteristic of a native enzyme capable of hydrolyzing *N*-acylhomoserine lactones, which are QS signaling molecules of different G(−) bacterial cells. The latter is confirmed by the effectiveness of the enzyme in combination with APM (Table 3), which provides a significant improvement in the antimicrobial effect of the use of Bacitracin in relation to G(−)-bacterial cells.

The calculated data showed that Bacitracin demonstrated high affinity values (strong interaction) to the surface of the enzyme dimer molecule (Table 1), which means that such a combination can be stable and maintained when used. Speaking about the stability of the combination, the following should be noted. Since the total area occupied by bacitracin molecules on the surface of the enzyme dimer molecule and the affinity of AMP to the surface of His_6_-OPH at pH 10.5 compared to pH 7.5 of the media used to form combinations were higher during their modeling, it was expected that at pH 10.5 the catalytic stability of the combination with bacitracin could be higher. However, experimental verification of this expectation showed (Figure 3) that a higher residual enzymatic activity is observed for the combination of His_6_-OPH/Bacitracin formed at pH 7.5. It can be assumed that, despite the fact that a greater surface coverage of the enzyme molecule leads to an increase in affinity when interacting in the studied combination of the enzyme with AMP, this negatively affects the enzymatic activity as a result of a decrease in the mobility of the His_6_-OPH molecule. Additionally, this probably leads to a decrease in the availability of its active centers for the implementation of a catalytic reaction. The presence of Bacitracin in the medium with His_6_-OPH did not have a noticeable effect on the optimum pH of this enzyme, which remained typical (Figure 3). The thermal inactivation of the enzyme in the presence of Bacitracin classically depended on the temperature of exposure to the combination of His_6_-OPH in AMP and the protein concentration itself.

A study of the antimicrobial activity of the combination of His_6_-OPH/Bacitracin against bacteria and yeast (Table 3) showed that the presence of the enzyme in the medium with Bacitracin can significantly reduce the inhibitory concentration of the latter and increase its effectiveness against G(−) bacterial cells. This result is consistent with the previously-obtained data for His_6_-OPH [4,5], when the presence of the enzyme in an environment of cells with antimicrobial substances increases the effectiveness of the antimicrobials’ action by reducing the development of QS and decrease the cell resistance.

A comparison of the effectiveness of Bacitracin action with other previously studied AMP variants in combination with His_6_-OPH showed that it is an order of magnitude more effective against G(+) bacterial cells than many analogues under similar conditions [4]. With respect to G(−) cells, the low effectiveness of Bacitracin is known, but in the presence of a combination with His_6_-OPH, its effect is significantly improved.

The results obtained for the first time in this work with yeast cells showed that the mechanism of action of Bacitracin on the synthesis of the NAG carrier actually makes it a universal antimicrobial substance. However, an even more detailed elucidation of the mechanism of enhancing the effect of Bacitracin on yeast cells in the composition of His_6_-OPH/Bacitracin, as it turned out (Table 4), can be continued and become a separate important study from a scientific and practical point of view. At the same time, new data obtained in this work on the possible objects of exposure to His_6_-OPH/Bacitracin and the level of its effectiveness can significantly expand the boundaries of the use of this composition. In particular, it may be interesting for use in various fields of medicine and veterinary medicine in the treatment of diseases associated with complications, in particular, with the appearance of secondary infections and the development of candidiasis and other fungal lesions in people with weakened immunity, in particular, after COVID-19, burns, after operations in diabetics, etc. Such studies may be associated not only with the study of the resulting combination of His_6_-OPH/Bacitracin, but also with the creation of other combinations of Bacitracin with new data. Although, it should be noted that to date His_6_-OPH is one of the best quantitative easing programs with respect to the diversity of lactone-containing molecules hydrolyzed by it [3,21], which was confirmed by the breadth of the spectrum additionally disclosed in this work.

It should be noted that the result was achieved in the work to improve the antimicrobial action of Bacitracin without its chemical modification, which many modern studies are focused on [10,11,12]. It turned out that it is possible to expand the spectrum of action of Bacitracin and enhance its effectiveness by simply mixing solutions of the enzyme and the antibiotic in order to obtain a good interaction between these components while preserving and even improving the characteristics of both of them. However, initially partners for such a combination can be found and analyzed in silico.

Talking about the practical potential of the results obtained, and taking into account the mechanism of antimicrobial action of Bacitracin in a composition with QQE, it seems appropriate to surface use of this composition for the treatment of various skin injuries in humans or animals, including burns [27,28], and for the development of improved wound-healing materials, to which the practical interest is high [5,29].

In addition to medical purposes, for the combination of His_6_-OPH/Bacitracin, its possible use can be assumed for the purpose of local suppression of methanogenesis in places of accumulation of decomposing debris, the intensity of which is caused by the formation of stable microbial consortia, which may include a significant number of different bacterial cells in the QS state [30], as well as archaea, sensitive to Bacitracin [31].

Another promising area of application of the His_6_-OPH/Bacitracin composition may be the local disinfection treatment of places where biofilms are formed, in particular, biocorrosive ones, which can lead to damage of expensive equipment, for example, pumping systems. Interest in compounds that can be used effectively to suppress biocorrosion does not decrease today, they are actively synthesized, but the main attention is paid to those variants of biocorrosion inhibitors that have an initial natural origin [32].

At the same time, the biocidal activity of the His_6_-OPH/Bacitracin composition in relation to systems with biocorrosive biofilms, as in this work, can also be evaluated using bioluminescent ATP-metry [32]. It should be noted that the effectiveness of the combination of His_6_-OPH/Bacitracin was established in relation to two individual cultures (*Rhodococcus* and *Pseudomonas* genera) (Table 3) from among the frequent aerobic heterotrophic bacterial participants-catalysts of biocorrosion processes, where the anaerobic agent is sulfate-reducing bacteria [33]. In this regard, it is clearly expedient to study the resulting combination in systems with a high risk of developing biocorrosion processes in order to inhibit the formation of biofilms, due to its wide range of antimicrobial action and the uniqueness of the mechanism of action of Bacitracin itself.

The possibility of spreading the applied approach, namely, combining QQE with antibiotics, to the development of highly effective antimicrobial drugs seems simple and attractive not only for use against bacteria, but also to cells of other microorganisms, including those involved in the formation of various heterogeneous consortia in the form of biofilms based on pro- and eukaryotes, in particular, bacteria and yeast of the genus *Candida* [34,35].

## 4. Materials and Methods

### 4.1. Computational Methods

The structure of His_6_-OPH dimer was prepared using known crystallographic structure of OPH (PDB: 1QW7) which was modified by His_6_-tag as described previously [7].

ChemBioDraw software (ver. 12.0, CambridgeSoft, Waltham, MA, USA) and ChemBio3D were used to create ligands (Bacitracin and lactones) structure and to apply energy minimization with force field MM2.

Adaptive Poisson-Boltzmann solver (APBS) and PDB2PQR servers (ver. 1.4.2.1 and 2.1.1, respectively, available at http://www.poissonboltzmann.org/, accessed on 18 August 2022) with PARSE forcefield and default settings were used to calculate the surface charge distribution of both His_6_-OPH and Bacitracin [36,37]. Obtained structures in PQR and PDB format were converted to the PDBQT format using AutoDockTools (as part of MGLTools ver. 1.5.6, available at http://mgltools.scripps.edu/, accessed on 18 August 2022) with atomic charges calculated with the Gasteiger-Marsili method [38].

AutoDockVina (ver. 1.1.2, available at http://vina.scripps.edu/, accessed on 18 August 2022), which includes several terms in its own scoring function (Gaussian, repulsion, hydrophobic, hydrogen bonding, and the number of rotatable bonds) was used for docking [39]. Two conformations of Bacitracin (at pH 7.5 and 10.5) and single conformation of fungal lactones (at pH 8.0) were docked to the surface of corresponding conformations of His_6_-OPH dimer obtained at the same pH values. That was made on a desktop computer equipped with Intel Pentium Dual-Core CPU E5400 2.7 GHz and 3 GB of available memory. Briefly, the grid box was approximately centered on the center of mass of the His_6_-OPH dimer. The size of the grid box was chosen so that the enzyme surface was within the box with an additional margin (overall volume was ca. 360 nm^3^; compared with the volume of the OPH dimer of less than 220 nm^3^, PDB number 1QW 7 can be viewed for more information). Calculations were performed with default program options. Based on our previous studies with His_6_-OPH and results obtained in the development of new effective antibacterials [4,21], the best 12 poses with minimal energy were selected.

The “*get_area*” function of PyMOL was used to calculate solvent accessible area occupied by Bacitracin on the surface of His_6_-OPH homodimer.

### 4.2. Enzyme Preparation and Determination of Its Activity

His_6_-OPH was isolated from biomass of developed *E. coli* SG13009[pREP4] strain, purified and characterized by previously published methods [40]. In short, protein concentration was determined by Bradford assay with Coomassie Brilliant Blue G-250 (Sigma-Aldrich, Darmstadt, Germany) and its purity was analyzed by sodium dodecyl sulfate polyacrylamide gel electrophoresis in 12% polyacrylamide gel using Mini-PROTEAN II cell (Bio-Rad, Hercules, CA, USA) followed by Coomassie Brilliant Blue R-250 (Sigma-Aldrich, Germany) staining. Enzymatic activity was measured using the Agilent 8453 UV-visible spectroscopy system (Agilent Technology, Waldbronn, Germany) equipped with a thermostatted analytical cell with 10 mM aqueous Paraoxon (Sigma-Aldrich, Darmstadt, Germany) solution at 405 nm in a 100 mM Na-carbonate buffer (pH 10.5). Concentration of His_6_-OPH was ca. 2.5 × 10^−9^ M in the reaction cuvette. One unit of enzyme activity (U) was defined as the quantity of the enzyme necessary to hydrolyze 1 μmol of Paraoxon per min at 25 °C. The purity and specific activity of His_6_-OPH preparation obtained (*M*_W_ ≈ 37 kDa) was ca. 98% and 6000 U/mg.

Combinations of Bacitracin with His_6_-OPH were prepared according to the following procedure: solution of Bacitracin at concentration of 0.8 g/L in 50 mM K-phosphate buffer (pH 7.5) or 50 mM Na-carbonate buffer (pH 10.5) containing 150 mM NaCl, were mixed with solution of 1 g/L His_6_-OPH in the same buffer at 1:1 (*v*/*v*) ratio and exposed for 30 min at room temperature. The final concentrations of His_6_-OPH and Bacitracin were 41 mg/L and 32 mg/L, respectively. After that enzyme activity of the combination prepared was measured as described earlier while varying Paraoxon concentration in a reaction mixture from 10 to 200 µM to determine catalytic characteristics.

The values of the Michaelis constant (K_m_) and the maximum rate of enzymatic reaction (V_max_) were calculated by hyperbolic approximation using the least squares method in Origin Pro (ver. 8.1 SR3, OriginLab, Northampton, MA, USA). The obtained K_m_ and V_max_ values were further used to calculate catalytic constant (V_max_/E_0_) and action efficiency constant (V_max_/(E_0_ × K_m_) values for enzymatic activity.

The effect of physico-chemical parameters on enzyme activity was tested. Kinetics of thermoinactivation was studied at 8 °C, 25 °C, and 37 °C and pH 7.5 by periodic sampling and measuring the residual activity. Dependence of enzyme catalytic activity on the pH was determined using buffers with overlapping pH values: 50 mM phosphate buffer (pH 6.0; 7.5), 50 mM phosphate/100 mM carbonate buffer (pH 8.0) and 100 mM carbonate buffer (pH 10.0; 12.0).

### 4.3. Determination of Antimicrobial Effect of Bacitracin and Its Combination with His_6_-OPH

50% inhibitory concentration (IC_50_) value of Bacitracin alone or in combination with His_6_-OPH (1 g/L) was determined as described previously [4] with minor modifications. For that, overnight bacterial cultures (*Agrobacterium tumefaciens* B-8833, *Escherichia coli* DH5α, *Pseudomonas* sp. 78G, *Bacillus subtilis* B-522) and yeasts (*Candida* sp., *Cryptococcus albidus*, *Kluyveromyces marxianus*, *Pachysolen tannophilus*, *Saccharomyces cerevisiae*, *Torulopsis* spp., *Trichosporon beigelii*) were used.

To accumulate biomass, the cells of bacteria *Agrobacterium tumefaciens* B-8833, *Escherichia coli* DH5α, *Pseudomonas* sp. 78G, *Bacillus subtilis* B-522, *Rhodococcus erythropolis* AC-1514D and *Rhodococcus ruber* AC-1513D were aerobically cultivated in the corresponding culture medium as described previously [3,5]. Cells of *Candida* sp., *Cryptococcus albidus*, *Kluyveromyces marxianus*, *Pachysolen tannophilus*, *Saccharomyces cerevisiae*, *Torulopsis* spp., and *Trichosporon beigelii* yeasts were grown in the medium with the following composition (g/L): glucose—10, yeast extract—5, tryptone—10, Na citrate—1, K_2_HPO_4_·3H_2_O—3, MgSO_4_·7H_2_O—0.5, ascorbic acid—3. The bacterial and yeast cells were cultivated using a thermostatically controlled Adolf Kuhner AG shaker (Basel, Switzerland) at 28 °C (for G(−), and yeasts) and 30 °C (for G(+)), with stirring at 150 rpm.

Cell growth was monitored with an Agilent UV-8453 spectrophotometer (Agilent Technology, Waldbronn, Germany) at 540 nm.

All microorganisms were separated after cultivation from the nutrition media by centrifugation. Further, the cells as suspensions in a saline (ca. (1 ± 0.1) × 10^6^ cells/mL) prepared on basis of 100 mM carbonate buffer (pH 8.0) with Zn^2+^ ions (10^−5^ M) to provide the His_6_-OPH conditions for activity realization were used in the experiments. The cells were exposed at room temperature for 24 h with Bacitracin added in a concentration range from 0 to 32 mg/L.

The concentration of intracellular ATP was determined using a standard ATP reagent (Lyumtek OOO, Moscow, Russia) and luciferin-luciferase method to evaluate the residual concentration of viable cells in exposed samples using published procedure [5]. The intensity of bioluminescence was recorded using a Microluminometer 3560 (New Horizons Diagnostic, Arbutus, MD, USA). The concentration of Bacitracin leading to 50% loss of ATP as compared to control cell samples without antimicrobial treatment was assumed as IC_50_.

All data are presented as means of at least three independent experiments ± standard deviation (±SD). Statistical analysis was realized using SigmaPlot (ver. 12.5, Systat Software Inc., San Jose, CA, USA).

## 5. Conclusions

Thus, in this study, a new effective combination of His_6_-OPH enzyme and such AMP as Bacitracin was obtained and the expediency of combining them in silico and in vitro was demonstrated. To the best of our knowledge, the antimicrobial activity of Bacitracin against yeast cells was demonstrated for the first time and an increase in its effectiveness in the presence of His_6_-OPH was established. The results of in silico studies once again confirmed the appropriateness of using computational methods as relatively fast and reliable way to predict effectiveness of such combinations of His_6_-OPH. These studies have allowed to obtain data according to which the reason for the success of the action of the His_6_-OPH/Bacitracin combination on yeast cells can be associated, as in the case of G(−)-bacterial cells, not only with the universal action of the antibiotic itself, but also with the ability of the enzyme used in the work to catalyze the hydrolysis of microbial lactone-containing molecules important for the metabolic and functional activity of microorganisms, as well as for their stable viability.

## Figures and Tables

**Table 1:** Values of affinity, net charge, and area of His_6_-OPH dimer surface occupied by Bacitracin (N = 12) at two pH values of medium. The colors in the Table correspond to the areas occupied by Bacitracin which are colored in Figure 2.

pH	Net Charge	Affinity (kJ/mol)				Area (%)	
		Mean	Median	Upper (Lower) Bounds	*p* Value	Near Active Sites	Total
7.5	−3.8	−24.1	−24.3 ± 1.2	−23.0 (−25.0)	<0.001	0.1	11.4
10.5	−7.4	−27.6	−27.2 ± 1.2	−26.8 (−28.3)		0.1	14.2

**Table 2:** Catalytic characteristics of His_6_-OPH in combination with Bacitracin and without it.

Enzyme or Complex	K_m_ (μM)	V_max_/E_0_ (1/s)	V_max_/(E_0_ × K_m_) (1 × 10^6^/(M × s)
His_6_-OPH	10.5 ± 2.2	5040 ± 340	480 ± 154
His_6_–OPH/Bacitracin	15.1 ± 3.3	6632 ± 856	449 ± 217

**Table 3:** Inhibitory concentration (IC_50_ *, mg/L) of Bacitracin against different microorganisms alone or in combination with His_6_-OPH.

Microorganism	w/o His_6_-OPH	with His_6_-OPH	Decrease of IC_50_ Due to the Use of His_6_-OPH/Bacitracin Compared to the Bacitracin Alone (Times)
Bacterial cells			
*Escherichia coli* (G(−)) **	1.94 ± 0.12	1.56 ± 0.18	1.3
*Pseudomonas* sp. (G(−))	52.23 ± 3.01	16.5 ± 1.46	3.2
*Agrobacterium* sp. (G(−))	21.19 ± 0.22	6.41 ± 0.21	3.3
*Rhodococcus* sp. (G(+))	3.05 ± 0.28	3.5 ± 0.31	0.9
*Bacillus subtilis* (G(+))	0.11 ± 0.01	0.11 ± 0.01	1
Yeast cells			
*Candida* sp.	17.83 ± 2.41	14.83 ± 1.51	1.2
*Saccharomyces cerevisiae*	14.52 ± 1.4	9.85 ± 0.61	1.5
*Cryptococcus albidus*	2.8 ± 0.15	2.89 ± 0.15	1
*Pachysolen tannophilus*	14.92 ± 1.01	1.75 ± 0.21	8.5
*Kluyveromyces marxianus*	10.21 ± 0.71	2.6 ± 0.16	3.9
*Torulopsis* spp.	31.47 ± 2.04	5.76 ± 0.05	5.5
*Trichosporon beigelii*	15.65 ± 0.75	3.98 ± 0.06	3.9

* The concentration of Bacitracin leading to 50% loss of intracellular ATP concentration in analyzed cell samples as compared to control without antimicrobial treatment was assumed as IC_50_. ** G(−)–gram negative bacteria, G(+)–gram positive bacterial cells.

**Table 4:** Values of occupied area and affinity of lactone molecules to the surface of His_6_-OPH dimer in the absence and in the presence of Bacitracin.

Lactones	His_6_-OPH without Bacitracin			His_6_-OPH with Bacitracin		
	Area, %		Affinity, (kJ/mol)	Area, %		Affinity, (kJ/mol)
	Near Active Site	Total	Median	Near Active Site	Total	Median
γ-butyrolactone	**1.1**	3	−16.3 ± 0.8	**1**	3.7	−16.1 ± 0.9
γ-heptalactone	**0.8**	3.1	−20.7 ± 1.3	**0.6**	3.5	−20.7 ± 2.0
γ-decalactone	**0.6**	4.9	−21.1 ± 1.6	**0.4**	3.3	−22.0 ± 1.5
Gluconolactone	0.1	6	−24.1 ± 0.8	0.1	4.6	−23.2 ± 1.2
Butyrolactone I	0.2	9.3	−30.3 ± 1.4	0.1	9.1	−29.5 ± 1.6
Multicolanic acid	0.6	6.6	−22.6 ± 1.5	0.1	4.8	−21.7 ± 1.3
Multicolosic acid	0.1	14.9	−21.3 ± 2.1	0.1	7.8	−20.7 ± 1.7
Multicolic acid	0.1	6.9	−23.6 ± 1.2	0.3	5.9	−22.2 ± 0.5

The bold corresponds to lactone molecules, for which the highest level of probability of hydrolysis by the His_6_-OPH.

**Table 5:** Comparison of the impact of peptides located on the surface of enzymes on their catalytic activity.

Enzyme; [Reference]	Peptide	* Interaction with the Active Site Residues	Effect on Catalytic Activity
Guanosine triphosphatase (GTPase) FtsZ; [22]	Temporin L	+	competitive inhibition
Cathepsin L (CatL); [23]	LL-37	+	competitive inhibition
Myeloperoxidase (MPO); [24]	Antioxidative peptide TEFHLL	+	inhibition
Angiotensin I-converting enzyme (ACE); [25]	Antihypertensive peptide FHAPWK	+	competitive inhibition
DNA gyrase and Lanosterol 14-alpha demethylase; [26]	Cyclo (Thr-Arg-Pro-D-Val-Leu)	+	competitive inhibition

* Symbol “+” means blocking of entrance to active site of the enzyme by peptide.

## Data Availability

The data presented in this study are available by request from the corresponding author.

## Supplementary Materials

The following supporting information can be downloaded at: https://www.mdpi.com/article/10.3390/ijms23169400/s1.
