# Supplementary material for: “Universal” Antimicrobial Combination of Bacitracin and His6-OPH with Lactonase Activity, Acting against Various Bacterial and Yeast Cells
Source: Int J Mol Sci. 2022 Aug 20;23(16):9400. doi: 10.3390/ijms23169400 (PMC9409362; doi:10.3390/ijms23169400)
Supplement: Supplementary file 1 [file ijms-23-09400-s001.zip › ijms-1858030-supplementary.pdf]

Supplementary Materials

International Journal of Molecular Sciences

“Universal” antimicrobial combination of bacitracin and His_6-OPH with lactonase activity, acting against various bacterial and yeast cells

Aysel Aslanli, Maksim Domnin, Nikolay Stepanov, Elena Efremenko*

Chemical Faculty, Lomonosov Moscow State University, Lenin Hills 1/3, 119991 Moscow, Russia
Correspondence: elena_efremenko@list.ru; Tel.: +7-495-939-3170; Fax: +7-495-939-5417

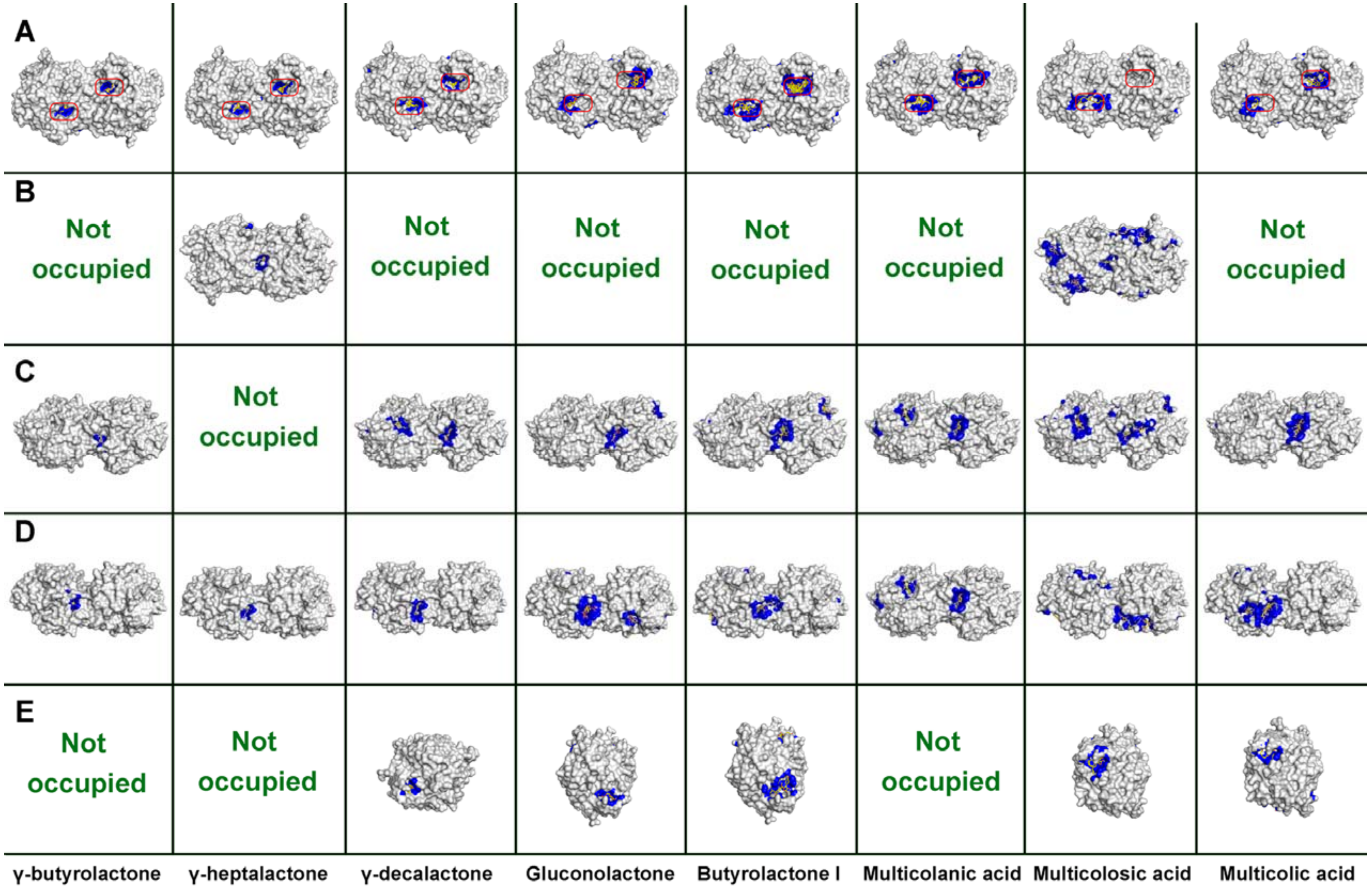

Figure S1. Computationally predicted localization of lactone molecules (colored yellow) on the front (A), back (B), above (C), below (D) and side (E) surface of dimeric His$_6$-OPH molecule (colored grey) at pH 8 in the absence of Bacitracin. The molecular surface corresponding to atoms located within 4 Å of any lactone atom is colored blue. The entrances to the active sites of His$_6$-OPH dimer are highlighted with red boxes.

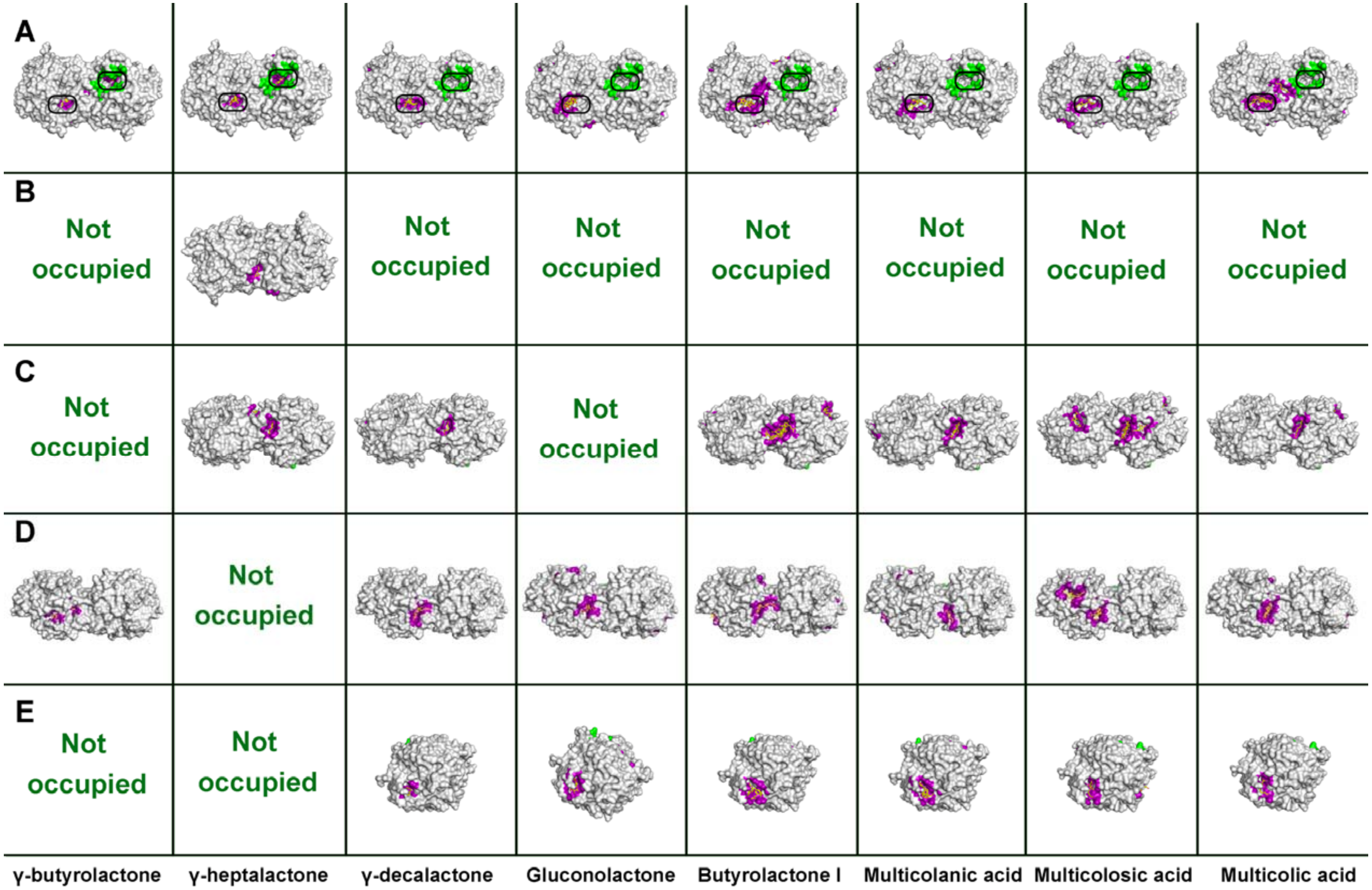

Figure S2. Computationally predicted localization of lactone molecules (colored yellow) on the front (A), back (B), above (C), below (D) and side (E) surface of dimeric His_6-OPH molecule (colored grey) at pH 8 in the presence of Bacitracin. The molecular surface corresponding to atoms located within 4 Å of any lactone atom is colored purple. The entrances to the active sites of His_6-OPH dimer are highlighted with black boxes.

Table S1. Calculated values of affinity of lactone molecules to the surface of His_6-OPH dimer in the absence and in the presence of Bacitracin.

**Lactones**	**Affinity, (kJ/mol)**				
	**pH**	**Mean**	**Median**	**Upper (Lower) Bounds**	**p value**
	His_6-OPH without Bacitracin				
γ-butyrolactone	8	-16.2	-16.3±0.8	-16.7 (-15.9)	0.671
	10.5	-16.4	-17±0.7	-17.1 (-15.5)	
γ-heptalactone	8	-20.7	-20.7±1.3	-22.2 (-19.5)	0.125
	10.5	-19.9	-21.9±1.5	-21.9 (-18.8)	
γ-decalactone	8	-21	-21.1±1.6	-22.1 (-19.4)	0.181
	10.5	-20.5	-20.9±1	-21.5 (-19.7)	
Gluconolactone	8	-24.1	-24.1±0.8	-25 (-23.5)	0.433
	10.5	-26.8	-26.2±1.6	-27.5 (-25.9)	
Butyrolactone I	8	-30.5	-30.3±1.4	-30.8 (-29.4)	<0.001
	10.5	-29.8	-29.3±1.8	-30.8 (-28.5)	
Multicolanic acid	8	-23.2	-22.6±1.5	-23.4 (-22.2)	0.011
	10.5	-22.3	-21.7±1.5	-22.5 (-21.3)	
Multicolosic acid	8	-21.9	-21.3±2.1	-22.1 (-20.6)	0.335
	10.5	-22.1	-22±1.2	-23 (-20.9)	
Multicolic acid	8	-23.6	-23.6±1.2	-24.2 (-22.6)	0.621
	10.5	-23.9	-23.6±1.5	-25 (-22.6)	
	His_6-OPH with Bacitracin				
γ-butyrolactone	8	-16.2	-16.1±0.9	-17.2 (-15.5)	0.953
	10.5	-16.3	-16.7±1.1	-17.2 (-15.1)	
γ-heptalactone	8	-20.7	-20.7±2	-22.5 (-18.9)	0.772
	10.5	-20.9	-21.1±1.1	-21.7 (-19.8)	
γ-decalactone	8	-21.9	-22±1.5	-22.8 (-20.5)	0.125
	10.5	-21	-20.7±1.3	-22.1 (-19.7)	
Gluconolactone	8	-23.2	-23.2±1.2	-23.4 (-22.2)	0.250
	10.5	-22.5	-22±1.5	-23.9 (-21.3)	

Butyrolactone I	8	-29	-29.5±1.6	-30.1 (-27.6)	0.303
	10.5	-29.7	-29.1±1.6	-30.7 (-28.5)	
Multicolanic acid	8	-22.1	-21.7±1.3	-22.6 (-21.3)	0.0658
	10.5	-22.2	-22±0.9	-22.9 (-21.3)	
Multicolosic acid	8	-21.2	-20.7±1.7	-23 (-19.8)	0.01
	10.5	-23.1	-22.8±1.6	-24.3 (-22.2)	
Multicolic acid	8	-22	-22.2±0.5	-22.5 (-21.4)	0.004
	10.5	-22.8	-22.8±0.6	-23 (-22.3)	
